# Adolescents’ Food Choice and the Place of Plant-Based Foods

**DOI:** 10.3390/nu7064619

**Published:** 2015-06-09

**Authors:** Hannah Ensaff, Susan Coan, Pinki Sahota, Debbie Braybrook, Humaira Akter, Helen McLeod

**Affiliations:** 1 Nutrition & Dietetics, School of Health & Wellbeing, Leeds Beckett University, Leeds LS1 3HE, UK; E-Mails: s.coan@leedsbeckett.ac.uk (S.C.); p.sahota@leedsbeckett.ac.uk (P.S.); d.braybrook@leedsbeckett.ac.uk (D.B.); h.akter@leedsbeckett.ac.uk (H.A.); 2 Health & Wellbeing Service, Leeds City Council, Leeds LS12 1DB, UK; E-Mail: helen.mcleod@leeds.gov.uk

**Keywords:** adolescents, food choice, plant-based foods, qualitative methods

## Abstract

A diet dominated by plant foods, with limited amounts of refined processed foods and animal products conveys substantial health benefits. This study sought to explore adolescents’ attitudes and perceptions towards plant-based foods. Semi-structured focus group interviews were conducted with adolescents (age 14–15 years) (*n* = 29) attending an inner city school in Yorkshire, UK. Using a grounded theory methodology, data analysis provided four main categories and related concepts revolving around adolescents’ perspectives on plant-based foods: food choice parameters; perceived drivers and benefits of plant-based foods; environmental food cues; barriers to plant-based food choice. In the emergent grounded theory, a clear disconnect between plant-based foods and the parameters that adolescents use to make food choices, is highlighted. Further, key barriers to adolescents adopting a plant-based diet are differentiated and considered with respect to practice and policy. The analysis offers a framework to remodel and re-present plant-based foods. In this way, it is proposed that a closer connection is possible, with consequent shifts in adolescents’ dietary behaviour towards a more plant-based diet and associated health benefits.

## 1. Introduction

Across Europe there are rising levels of obesity among children and adolescents; in some states up to 44% and 38% of boys and girls, respectively, are overweight (including obese) [1]. The prevalence increases with age, such that the majority (52%) of European adults are overweight or obese [2]. This is the culmination of dramatic increases seen across Europe since the 1990s [2]. In the UK, obesity has almost doubled in the last twenty years, and currently stands at the second highest in Europe at 26.1% [2]. For children in the UK, the picture is particularly bleak, with combined overweight and obesity prevalence at 34% and 37% for 11 to 15-year-old boys and girls, respectively [3]. 

Adolescents’ diet in the UK typically falls short of dietary recommendations with excessive intakes of saturated fat and sugar, as well as low fruit and vegetable consumption [4]. The majority of British 15-year-olds do not meet the recommended five portions of fruit and vegetables every day; further, 64% do not eat fruit daily and 63% do not eat vegetables daily [5]. Adolescents’ diet is also generally marked with poor food choice, including a high proportion of nutrient-poor and fast foods [6,7,8]. The implication of this on public health is well-documented [9,10,11]; and the high burden of disease due to unhealthy dietary patterns has been acknowledged by World Health Organisation European Member States, with particular concern for childhood obesity and overweight [12].

A plant-based diet, with an emphasis on plant foods (vegetables, fruit, pulses, grains and nuts) and limited amounts of refined processed foods and animal products (meat, chicken, fish, dairy and eggs), conveys substantial health benefits [13,14]. A plant-based diet can protect against certain chronic diseases, such as heart disease [13,15], diabetes [15,16,17], certain cancers [15,18,19] and obesity [20]. Evidence also suggests that including fruit and vegetable consumption within a weight management strategy is particularly effective [21]. It has been acknowledged that UK public health would benefit if the public consumed less meat and dairy and more fruit and vegetables [22]. Modelling has also demonstrated that dietary shifts involving meat reduction can convey substantial public health benefits to a nation, in terms of deaths delayed or averted [23].

Food habits and eating behaviour are learnt in childhood and adolescence; they also tend to persist into adult life [24,25]. Thus, a plant-based diet during adolescence is not only an important dietary quality indicator, but can also promote healthy food choice later on in life. As adolescents exercise a greater degree of autonomy over food choices, opportunities to intervene and actuate change should be explored. Given the potential benefit of a plant-based diet, there has been little research on the requirements and processes involved in shifting diets towards more plant-based eating [26,27]. Indeed, there is a limited evidence base for such policies [28], and this extends to adolescents [27], where knowledge of attitudes regarding shifting diets is also lacking. 

This qualitative study sought to examine adolescents’ attitudes and perceptions towards plant-based foods, from their perspective and within the context of their food choice parameters. A key motivator for this work was to provide some evidence for the best options for orchestrating a shift in adolescents’ dietary patterns. A grounded theory methodology [29,30] was chosen for this study. This approach involves systematic and iterative analysis of collected data, and results in a theory that has emerged from the data itself. The methodology was considered the most appropriate to develop a model of adolescents’ perspectives of plant-based foods. 

## 2. Experimental Section

Ethical approval for this study was granted by Leeds Beckett University’s Faculty of Health and Social Sciences’ Research Ethics Committee (reference number 2013-14 LREC 02; 5 November 2013).

### 2.1. Study Participants

The participants were adolescents (age 14–15 years) attending an inner city school in Yorkshire. The school was a large secondary school with approximately 1100 students (age 11–18 years) on roll. The school had a higher than average Free School Meal (FSM) profile (the FSM programme is a national programme providing a free school meal for students of low income families); 33% of students were eligible and claimed FSM—compared to the national average of 15% in England [31]. The percentage of students with English not as a first language (26%) was also higher than the national average (14%) [31]. 

Participants were recruited via information sheets and introductory letters. All Year 10 (age 14–15 years) students were invited to take part in the study. This particular year group was selected as it corresponds to mid-adolescence; it was also an appropriate year group from the school’s perspective, with respect to avoiding disruption to examination curriculum time. Out of those students consenting to take part, the school leadership selected a representative sample of thirty students (16 boys, 14 girls), based on school held data and across three key contextual factors: FSM, gender, and academic achievement. 

### 2.2. Focus Group Interviews

Focus group interviews were selected as the means of data collection. This was to promote participant discussion and to collect rich data, in order to enable valuable insights. The research team conducted all focus group interviews during school time and on site during the academic year 2013–2014. Informed consent was completed prior to data collection, and data anonymisation and storage measures were preserved throughout the study. No one declined to take part in the study, however one student was absent.

The focus group interviews were semi-structured and limited to five participants per group in order to allow flexibility, encourage free discussion and provide enough opportunity for participants to contribute and explore relevant issues. The interviews were moderated using a prepared schedule of topics. Key questions were followed by probes, as required, to promote discussion and exploration. The schedule of topics focused on participants’ attitudes, perceptions and experiences of plant-based foods. Initially, the questions covered how students choose what to eat and the relative importance of food choice parameters, before progressing onto plant-based diets. The interviews incorporated a number of activities to encourage engagement and form the basis of subsequent discussions. These included an introductory activity where students were asked to write down and then describe their lunch choice from the previous day. A second activity entailed a shopping basket exercise, where students were shown photographs of four pairs of comparable shopping baskets (one with a stronger emphasis on plant-based foods); students were asked to select their preferred option and then consider their reasoning, and describe and discuss these. The concept of a plant-based diet was then introduced and explained; it was described as a diet with an emphasis on plant foods (vegetables, fruit, pulses, grains and nuts), limited amounts of animal products (meat, chicken, fish, dairy and eggs) and limited refined processed foods. It was distinguished from an exclusive vegan or vegetarian diet. A plant-based diet was also described within the context of a spectrum of diet using different examples, e.g., omnivore, vegan. Participants were asked how their diet corresponded to this spectrum of diet and to approximate its plant-based component. At the end of all focus group interviews, participants were asked if there was any additional information not already discussed that they thought was relevant or important to include in the analysis. A prepared schedule of topics was used for all interviews; however, discussions were not constrained to a particular format, and free discussion was encouraged as and when relevant issues arose. Following each focus group interview, the research team recorded key ideas and thoughts (which were included in the subsequent data analysis). The schedule of topics was also considered between successive interviews to ensure the inclusion of evolving concepts. In total, there were six focus group discussions, with the majority lasting between 40 and 50 min; this proved ample for theoretical saturation (the point at which no new conceptual insights emerge, and the relationships between categories are well defined).

### 2.3. Data Analysis

Audio recordings of the interviews were made with audio software GarageBand (Apple Inc., Cupertino, CA) and a microphone on a MacBook Pro. All audio files were transcribed by one researcher and checked by another researcher (present at the focus group interviews). The audio files were transcribed using a protocol, and with an emphasis on denaturalised transcription. This was to provide an emphasis on the content and essence of discussions, and in order to connect with participants’ viewpoints and perceptions. This approach was selected to suit the study’s grounded theory methodology. 

The transcripts and material produced from the focus group activities, as well as the memos from the interviews provided the data for this study. Software (NVivo10, QSR International, Victoria, Australia) was used to help with the overall data management and data analysis. 

Data were analysed to capture participants’ attitudes and perceptions around plant-based foods and food choice. During the data analysis, emergent themes and concepts were recorded and explored using memoing (reflective memo writing to capture interpretative thoughts and questions about the data). Initially, the transcripts and study objectives were examined to draw up a list of categories, for which representative nodes were created. Two researchers analysed and coded the transcripts independently. Systematic discussion between the researchers on emergent nodes and concepts allowed discrepancies to be reviewed in order to reach consensus. The data were coded in an iterative fashion, with the overall goal of developing a descriptive theory of the relationship between the emergent themes and patterns. Queries, models, coding stripes and node coding reports were generated to help with the development of emergent theories, which were themselves examined against existing theories and the collected data. In this way a list of finalised nodes, concepts and categories emerged. The relationships between the final categories and concepts, as well as their relative influences were then defined to form the grounded theory. Coding was completed for all transcripts, and done so beyond the point at which no new conceptual insights emerged and theoretical saturation was felt to have been reached, in order to substantiate findings. The constant re-evaluation of the data, systematic discussion of emerging categories and concepts, as well as memoing, were all used to control for researcher bias. 

## 3. Results

Data analysis provided four main categories (food choice parameters, perceived drivers and benefits of plant-based foods, barriers to plant-based food choice, and environmental food cues) each with their related concepts, presented in Table 1. The following account provides a consideration of each concept, and the grounded theory that emerged from their relative influences and interrelationships.

**Table 1:** Categories and concepts.

**Food choice parameters**—“*you want to get something that actually tastes good*”
Food taste, appearance
Personal food history, habits and familiarity
Peers
Convenience
Price
**Perceived drivers and benefits of plant-based foods** —*why “others” choose plant-based foods*
Animal welfare, dislike of meat
Health, variety, freshness
**Barriers to plant-based food choice**—*“it’s not the kind of food I eat”*
Food neophobia and fussiness
Effort to prepare
Perceptions of tastiness
Food identity
Confusion, including around health benefits
**Environmental food cues**
Food visibility, smell, accessibility
Dining environment, atmosphere

### 3.1. Food Choice Parameters—“You Want to Get Something That Actually Tastes Good”

Students adopted several parameters when they chose food; some were based around food attributes, whilst others related to their own food habits and experience. Each of these influenced the food selections made and typically one or two were key, depending upon the particular food. 

#### 3.1.1. Food Taste, Appearance

Students acknowledged that taste was of primary importance when choosing what to eat.

*… “taste” first ’cause you want to get something that actually tastes good.*

[ST27]

*It’s gotta taste good.*

[ST21]

*As long as it tastes good, then it’s alright.*

[ST23]

Sometimes a single component or ingredient was a key factor in deciding whether or not to choose a food, e.g., *“I don’t like cauliflower” [ST28], “I don’t like prawns” [ST2].* Often this was related to taste.

*I don’t really have beef … just the taste … don’t really like it.*

[ST18]

*I just like prawns more than veg … way more! … it has a nice taste.*

[ST27]

Other times, there was a more general response to a group of foods, *“Yeah, I’m not really big on vegetables” [ST17]* and *“I’m not really keen on veg …” [ST8].*

The appearance of food was another key parameter, and there was a general feeling among students that *“it has to look good for you to want to buy it” [ST8].*

#### 3.1.2. Personal Food History, Habits and Familiarity

As well as the food itself, it was apparent that students’ experiences strongly influenced their food preferences, and responses to particular foods. In this way, students’ personal food history, habits and familiarity played a key role in determining food choice. Students were very keen to stick to familiar foods or foods that looked like the kind of food they knew.

*… it looks more [like] the food I eat.*

[ST9]

*I like to stick with foods that I know I like …*

[ST4]

*Looks like … something that I’d go for …*

[ST8]

Students also expressed a preference for foods that they had a connection with. Often, as would be expected, this connection emanated from food provision at home.

*I don’t know … I usually have it at home so I think “yeah, it’s really nice” …*

[ST6]

*Because I like prawns and my mum makes stuff like that all the time … and I like that sort of stuff.*

[ST28]

#### 3.1.3. Peers

During the interviews, adolescents discussed the influence of peers on food choice; there was not however a general consensus on this. Some students acknowledged their significance: *“I get whatever my mates get*” *[ST15]*, whilst others were definite in making their own decisions: “*’cause they [friends] might have different taste than you” [ST4]*; *“’cause I don’t copy my friends … ” [ST13]*.

Whilst students may not have wanted to be perceived to be copying their friends, the influence of peers was evident in earlier discussions regarding taste. One student commented on how, if a friend had tried a particular food and reported it as “tasty” then this would encourage them to try it (intimating the role of peers and also reinforcing the significance of taste when it comes to food choice).

#### 3.1.4. Convenience

During the focus group interviews, adolescents spoke of the importance of convenience when it came to selecting food—convenience both in terms of ease of food preparation as well as portability of purchased food, *i.e.*, “grab-and-go”. This was especially the case when time was limited or when they were keen to take their food away. This student explains how in the school canteen for example, food choice may be dictated by other pulls:

*Yeah, it’s easier to [grab something] … easily and quicker and then you can just go straight out … ’cause I play football at break …*

[ST18]

Another student explained how having a portable meal made it easier to socialise:

*… and then “convenience”, ’cause you can just sit with your friends you know …*

[ST27]

When considering food choice outside the school dining environment, ease and convenience were relevant to students. Interestingly this featured most significantly when meals were specifically destined to be quick meals.

*I don’t know … when I’m cooking pasta, I just don’t like standing there for long. I don’t really sit in there chopping … I’m weird with my pasta, I like it easy and fast …*

[ST16]

*And then, there’s some other things like … I like Pot Noodle* (*a brand of instant noodle snack in a plastic pot, prepared by adding hot water) at home with something … like if I can’t be bothered making … so I just a grab a Pot Noodle from [the] shop.*

[ST6]

#### 3.1.5. Price

Students were clear about the inclusion of price within food choice parameters. However, price was one of many parameters, and one student described the importance of price relative to taste:

*Don’t really matter about the price ’cause if you’re buying like a pizza, it’s 50p and it’s terrible, and then if you buy like a £1.50 pizza and it’s tasty … I’m gonna buy the delicious pizza everyday.*

[ST6]

Overall, adolescents felt that the importance of price varied depending on the eating occasion and location. In particular, price became less important in the school canteen where students felt secure in knowing food prices would be within certain limits. When eating out however, price was more pertinent, and students were very aware of the need to consider price and value for money.

*Mostly when I go out, I’m more scared of the price than my taste, so price will be first [consideration] … when you go outside with your mates, you spend all [money] on a meal but you never finish it.*

[ST16]

### 3.2. Perceived Drivers and Benefits of Plant-Based Foods—Why “Others” Choose Plant-Based Foods

Adolescents had a distinctly “onlooker” perspective when considering plant-based foods. They had perceptions about the drivers and benefits for adopting a plant-based diet, and crucially this was seen exclusively with respect to “others”. The drivers mainly revolved around animal welfare and dislike of meat, whilst the benefits mainly came down to health, variety and freshness. Adolescents also displayed incomplete and superficial knowledge of the merits of a diet with limited refined and animal products but high in plant-based foods. They also generally had limited knowledge of and contact with people adopting such diets.

#### 3.2.1. Animal Welfare, Dislike of Meat

In discussions with students, there was a general association of plant-based diets exclusively with vegetarianism. This was despite an introduction to the concept of a plant-based diet within the context of a spectrum of diet, as well as a discussion of participants’ own diets and their relative plant-based components. The main drivers identified by adolescents revolved around animal welfare, and tended to focus on meat. Generally, students associated these food choices with people who: “*wanted to become a vegetarian … ’cause they really like animals … or they might have gone off meat or something” [ST6];* or *“don’t like eating meat and they think it’s cruel against animals and stuff … like some people think it’s like horrible to eat other animals” [ST13].*

Other students expressed similar reasoning for adopting a plant-based diet.

*Might just be a lifestyle … like they just might not like [meat] … the look of it or how it smells.*

[ST14]

*Maybe they had a bad experience with meat.*

[ST12]

#### 3.2.2. Health, Variety, Freshness

Whilst adolescents did not refer to the health benefits of plant-based foods *per se*, there was an acknowledgement and widespread association of vegetables and fruit with healthy eating.

*I think veggies are healthier and better food for you.*

[ST18]

*… and you know vegetables [are] good for you.*

[ST6]

*… if you’re eating a lot of like veg and fruits and that like … then obviously it’s healthy.*

[ST13]

A minority of students also specifically referred to wholefood/wholemeal options as healthier alternatives. Students also associated cooking from scratch with healthy eating, compared to processed and refined foods.

*It looks more like healthy and like … you make it yourself, it'll be like more fresh …*

[ST7]

*… the ingredients look more fresh in this one … because it’s fresh and not like mashed into a jar.*

[ST6]

During the focus group discussions, students conveyed a general feeling that plant-based foods offered a greater degree of variety and freshness, which may influence food choice especially when shopping:

*… just like the colours are more bright … so like when I went shopping, I would see that first, before I would look at that … it’s more attractive.*

[ST7]

### 3.3. Barriers to Plant-Based Food Choice—“It’s not the Kind of Food I Eat” 

Throughout the focus group interviews, various barriers specific to the adoption of a plant-based diet emerged from the discussions. It became apparent that students’ perceptions around plant-based foods, as well as their general food choice parameters, diminished the potential of their adoption of a plant-based diet. These barriers included food neophobia and fussiness, food identity, perceptions about the effort to adopt a plant-based diet, perceptions about the tastiness of plant-based foods, and confusion about health benefits.

#### 3.3.1. Food Neophobia and Fussiness

In tandem with the importance of familiarity in making food decisions, adolescents conveyed a general ethos of “*I like to stick with foods that I know I like” [ST4]*. Students also expressed a reluctance to try new foods.

*Probably wouldn’t try it …*

[ST22]

*I’d give it a little try but I wouldn’t eat it all … [only] taste it.*

[ST22]

Overall, students expressed a general *“I’m not really big on vegetables” [ST17]* attitude. When considering the wholefood aspect of plant-based foods, there was a general resistance to these features, despite students’ ready acknowledgement of their health benefits:

*I don’t like wholemeal pasta, I like the normal one …*

[ST19]

*It’s like … you can proper feel the grains of the wheat in it and it doesn’t even taste good … it tastes like cardboard …*

[ST27]

*I’d still prefer the first [non-wholemeal] one … don’t like wholemeal.*

[ST16]

#### 3.3.2. Effort to Prepare

As described within the previous category, students associated cooking from scratch with healthy eating. However, on the whole they did feel that the effort required for this was probably prohibitive. One student added though, that that would depend upon the end result in terms of what it tasted like—reinforcing taste as a significant food choice parameter, as previously described:

*… ’cause like if I’m cooking it and it tastes nice afterwards, it’s alright … it’s like … it’s worth my time.*

[ST7]

#### 3.3.3. Perceptions of Tastiness

During the focus group interviews it became apparent that there was a general feeling among adolescents that non plant-based food was more likely to be tasty because *“meat has more flavour in it” [ST28]* and “*looks like it’ll have more flavour” [ST27].* This links closely with adolescents’ focus on missing meat in plant-based foods, as revealed in their perceptions of the drivers for plant-based diets. Given the importance of taste within adolescents’ food choice parameters, adolescents’ perceptions of the tastiness of plant-based foods is significant.

#### 3.3.4. Food Identity

Associated with the concept of food habits and personal food history described in an earlier category, students did not feel any connection with plant-based foods, instead referring to it as being *“not the kind of food I eat” [ST14]* or *“for vegetarians” [ST6].* This distinct separation from plant-based foods reflects the “onlooker” perspective seen in the previous category, whereby the perceived drivers and benefits of plant-based foods were relayed with respect to “others”. 

#### 3.3.5. Confusion, Including around Health Benefits

Adolescents displayed a general understanding about healthy eating, a balanced diet and needing a *“variety of things to be healthy” [ST8]*. Some of the students interviewed showed a personal interest in healthy eating, and others acknowledged healthy eating to be important for some of their peers. In particular, this was discussed with reference to the potential impact of a healthy diet on sports performance. The level of understanding and appreciation however, varied between them. Interestingly, one student pointed out how fat, sugar and salt influenced how the food tasted:

*… and then, that’s really tasty ’cause it’s loads of fat and sugar and salt or what-not in it.*

[ST6]

As previously noted, and despite an introduction and consideration of plant-based diets during the focus group interviews, adolescents associated plant-based diets exclusively with vegetarianism. Common to all focus group interviews, there was a universal perception that meat was a prerequisite for a healthy diet.

*I think you need like meat and stuff for like protein and calcium … and stuff like that … not just vegetables and stuff.*

[ST7]

*I think you need like all your vitamins and minerals and all that … not just all vegetables.*

[ST9]

Students perceived a plant-based diet as *“unhealthy” [ST21] “‘cause you have to have it [meat] in your diet” [ST28].* Tied in with this perception, was that a plant-based diet would be detrimental *“’cause you need protein in your body” [ST17]* and that a plant-based diet *“is a bit healthy but not really … you need a lot of protein in your body to help you …” [ST18].*

Students also felt that eating fruit and vegetables was healthy up to a point. There was also an association between avoiding meat and not having enough food, as this student explained:

*… if you eat like a little amounts of veg and fruit and no meat, then I think that could be like critical for some people like … so basically not having enough food and stuff like that …*

[ST13]

### 3.4. Environmental Food Cues

Adolescents were not overtly aware of the influence of food cues and simple heuristics when it came to making daily food choices. Whilst environmental food cues (food visibility, smell, accessibility, dining environment, atmosphere) are significant not only in the food choice behaviour but also in the potential opportunities that heuristic processing and food choice architecture provide for the promotion of foods, the closest students came to recognising this was with respect to how food appeared or was packaged. 

### 3.5. The Grounded Theory 

**Figure 1:**
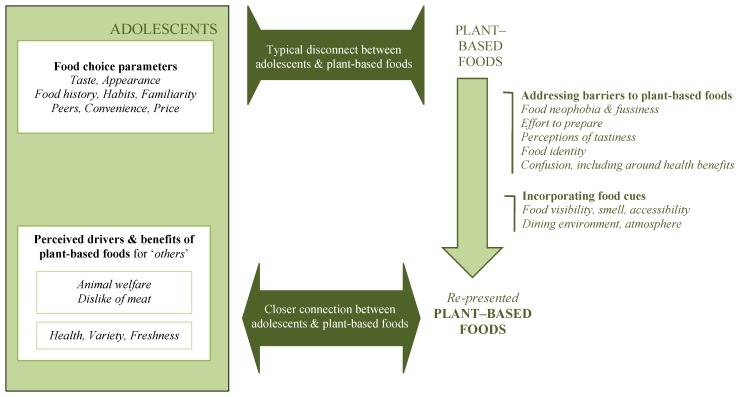
Adolescents and plant-based foods.

Data analysis resulted in a grounded theory where the disconnect between plant-based foods and adolescents’ food choice parameters is fundamental. The interrelationships and relative influence of the categories and concepts is modelled in Figure 1.

In making food choices, adolescents utilise parameters such as taste, habit, familiarity and convenience, and are heavily influenced by food cues, such as visibility and accessibility. Their perceptions about the drivers and benefits of a plant-based diet are with respect to “others”. Typically, adolescents do not encounter plant-based foods that correlate with their food choice parameters. This disconnect is fuelled by a number of factors such as food identity and food neophobia. However, these same barriers to plant-based foods, as well as adolescents’ general food choice parameters, and environmental food cues provide an opportunity and a framework to remodel and re-present plant-based foods, in order to promote a closer connection between adolescents and plant-based foods, and ultimately a shift in their dietary patterns. 

## 4. Discussion

In this analysis, adolescents’ food choice emerged as a product of many influences, including taste and appearance, as well as other socio-cultural factors informing social norms. Many of the emergent concepts (taste, convenience, appearance, price) concur with previous studies on adolescents [32,33,34]. Further, adolescents’ reported emphasis on taste as a critical factor correlates with previous work [32,35,36,37]. 

Also, in line with existing literature [38,39], students’ experiences and food history were found to influence their food preferences. Students quite often referred to their food identity when separating their food habits from plant-based food. This correlates with previous research on individuals’ personal systems of food choice and self-designated food identity [39]. 

Adolescents in this study had a superficial and incomplete knowledge of plant-based diets and the health benefits of plant-based foods. So whilst these attributes might otherwise encourage their consumption, they were poorly understood.

The findings also revealed perceptions with respect to the tastiness of plant-based foods, and a preference for other foods—notably meat. This taste differential in favour of other foods over healthier alternatives concurs with previous research [32,36,40], as does the taste barrier against plant-based foods [41]. These perceptions, in particular those relating to meat, as well as the requirement for more information, mirror findings in the limited existing literature [41,42,43] on barriers to the adoption of plant-based foods. 

### 4.1. Implications for Practice and Policy

In establishing insights into adolescents’ attitudes and perceptions towards plant-based diets, application of this knowledge is required in order to develop strategies to shift adolescents’ diets towards a higher proportion of plant-based food. 

There is substantial unrealised potential in doing so and the following account outlines some of the implications on policy and practice, which have been framed within the study’s emergent model. The proposed strategies themselves are based on adolescents’ food choice parameters, emergent barriers to plant-based foods, as well as an exploration of opportunities afforded by the food choice architecture. All the strategies themselves have potential barriers, e.g., cost, time and practicality—however the implications vary in terms of their resource outlay, and strategies should be tailored for the particular setting and consideration.

There is a need for policy to reflect and relay the significant health benefits afforded by plant-based foods, and all of the strategies described are contingent on policy will and change. 

#### 4.1.1. Familiarity and Plant-Based by Stealth

The study’s results underscore the importance of familiarity on food choices made by adolescents. Caterers, particularly in schools, therefore have the opportunity to develop a broad range of familiar foods with a high plant base. The reformulation could aim to increase the plant-based content of familiar foods, by reducing and/or substituting animal-based ingredients. It is proposed that this focus on the foods themselves rather than a label with connotations of plant-based, e.g., a “chilli” rather than a “vegetarian/vegetable chilli”, would ensure that available plant-based offerings are more in line with adolescents’ current eating habits and resonate with their food identity. Although this “plant-based by stealth” approach, in the short term, would not alter adolescents’ attitudes towards plant-based foods, it is a distinct opportunity with the potential to alter adolescents’ consumption of plant-based foods. In conjunction with addressing the familiarity aspect, there is a need to develop plant-based foods that respond to adolescents’ preferences for the structure and taste for meat. In particular, within adolescents’ food choice parameters, taste and appearance were prime, and as such, efforts to maximise these aspects of plant-based foods should be considered. Similar recommendations have been previously reported [32,43].

#### 4.1.2. Health and Freshness

Some students were interested in healthy eating, especially with respect to sports performance; this is in line with a recent study revealing Ecuadorean adolescents to report positive attitudes towards healthy eating [36]. Interest in healthy eating varied between students in this study; such variation has been seen previously in US adolescents [35,37], as has adolescents’ reluctance to be seen to be interested in healthy eating [44]. Nevertheless, there remains an argument for health attributes to be exploited to promote plant-based foods to those adolescents interested in healthy eating. This is the case particularly with those students personally interested in healthy eating, and the reciprocal benefits linked to sports and fitness. Likewise, the “fresh” aspect of plant-based foods as opposed to processed food, should be promoted. In tandem with this, the vibrant colour afforded more readily with plant-based foods, and recognised by adolescents in this study, offers good opportunities—especially given the significance of appearance within adolescents’ food choice parameters. 

#### 4.1.3. Education

In this study, adolescents displayed superficial knowledge of a plant-based diet and the health benefits of plant-based foods. They also perceived meat as a nutritionally necessary food—this has been shown previously in relation to perceived barriers to plant-based diets [43]. Nutrition education promoting a deeper understanding of a plant-based diet (including its distinction from a vegetarian diet) is therefore recommended, to enable more informed and deliberate choices in favour of plant-based foods. This may be of particular relevance to those students explicitly interested in healthy eating (especially with respect to sports). A focus on health benefits has been previously recommended in the promotion of plant-based foods to adults [41]. 

Sustainable diets of reduced environmental impact (in terms of greenhouse gas emissions and land use) lean strongly towards plant-based foods [45,46,47], and it has been shown that, with respect to environmental impact, the most relevant dietary distinctions are those between animal-based versus plant-based diets [48]. In this study, concepts around environmental issues and sustainability did not surface in discussions, and even when prompted, participants did not associate a plant-based diet with its environmental benefits. Previous research has shown that adolescents’ pro-environmental attitudes significantly predict pro-environmental behaviour, with environmental knowledge being a significant moderator [49]. This points to the value in providing opportunities for learning about food production and in particular, the environmental arguments in favour of plant-based foods. The value of adolescents knowing about the environmental impact of food production practices has been reported previously [50]. Knowledge alone however, is not enough; a perception that a change is needed is a key requirement for initiating dietary change [51]. It is proposed that complementary efforts to increase exposure to plant-based foods and education can diminish barriers and change the context in which adolescents perceive plant-based foods.

#### 4.1.4. Choice Architecture

The influence of choice architecture on adolescents’ food choice should not be underestimated. A recent review suggested the interaction between food preferences and the environment in which these are expressed, to be critical [52]. Accessibility of plant-based foods in particular, has a role to play. This aligns with the current policy priority of increasing the availability of healthy options [52], and previous research has shown how manipulating the choice environment can promote the selection and consumption of “healthy” foods [53,54,55]. There are therefore opportunities to incorporate such changes in order to nudge adolescents towards more plant-based food choices. In particular, the school dining environment provides a good setting for this work; students often referred to having to make a time-pressured decision—which lends itself well to automatic decision making and a reliance on heuristic devices [56] and consequently nudging. In addition, secondary schools quite often employ a “stay on site” policy during lunchtime, and again this restriction works particularly well with nudge strategies. This all strongly points to the potential of food choice architecture and the clear role it can play in changing adolescents’ food choice behaviour. Specifically, labelling and the use of descriptive names can be used to encourage healthier food choice behaviour, e.g., labelling pasta as “fresh Italian pasta with creamy tomato sauce”, rather than simply “vegetarian pasta”. This has been seen previously where foods are accorded attractive names [57], and with respect to plant-based foods—the freshness and health benefits can be utilised. In conjunction with this, nutrient profiling and labelling of foods with nutrient scores could help to promote plant-based foods, thereby enabling students to choose between items within the same food categories, *i.e.*, to “change up” their selection. The role of nutrient scoring tools in a healthier environment has been previously advocated [12]. 

As well as labelling, accessibility can be adjusted by more prominent positioning of plant-based foods. Previous research has shown how food choice can be changed by the position of foods, e.g., first in the line of foods available, or near the tills [53,54]. Similarly, adolescents’ predisposition for “grab-and-go” foods [7] can be applied to promote the uptake of plant-based foods, e.g., in providing meals in a portable disposable bowl. In this way, it is proposed that plant-based foods are more accessible to adolescents and likely to become part of their daily food habits. 

All the strategies proposed evolve from the attitudes and perceptions of adolescents, outlined in this study; further consideration of the social, cultural and economic implications is required. 

This study contributes to the literature on food choice and in particular, that related to adolescents. It provides rich insights into adolescents’ perspectives with respect to plant-based foods and crucially, the potential to shift adolescents’ food choice behaviour towards more favourable options. The presented grounded theory model portrays the clear disconnect between adolescents and plant-based foods. The model also contributes to the development of informed strategies around food choice, and more specifically outlines appropriate and informed action to engage the adolescent population and enable them to move towards a more plant-based diet.

### 4.2. Strengths and Limitations 

The study’s strength comes from its specific focus on adolescents’ perspectives towards plant-based food choice. Valuable insights into adolescents’ food choice parameters were revealed through the activities of the focus group interviews, in particular the shopping basket activity. The emergent model provides a framework for efforts to effect shifts in adolescents’ dietary patterns. 

Notwithstanding these strengths, the limitations of the study should be acknowledged. In particular, the selection bias (self-selection and then by the school) should be recognised. Likewise, the subjective nature of the process for participants and the data collected, is an important consideration in studies of this kind—in particular the recollection bias and self-report aspect. Whilst the adolescents in this study were not considered atypical, all participants were 14–15 year olds attending the same secondary school—and the findings should be interpreted within the particular context of the students and school, and so are not automatically extrapolatable. Further exploration with other samples of adolescents should be pursued. Another limitation to consider is the relative willingness of participants to freely and openly discuss relevant issues in a group environment, and this was evidenced by different students participating to differing levels in the focus group interviews. 

## 5. Conclusions 

This qualitative study has provided rich insights into adolescents’ perceptions of plant-based foods, all with respect to their food choice parameters. It has also highlighted the disconnect between adolescents and plant-based foods. The grounded theory portrays the complexities involved and the opportunities to remodel and re-present plant-based foods in order to shift adolescents’ diets. The emergent model provides a framework for exploring such strategies. 

Given the acknowledged challenge of adolescent obesity and the accompanying suboptimal dietary intake, there is a growing need to orchestrate efforts to improve adolescents’ dietary behaviour, based on their food choice parameters, as well as opportunities afforded by food choice architecture.
